# New Appliances and Things Medical

**Published:** 1902-08-23

**Authors:** 


### NEW APPLIANCES AND THINGS MEDICAL.

[We shall be glad to receive, at our Office, 28 & 29 Southampton Street, Strand, London, W.C., from the manufacturers, specimens of all new preparations and appliances which may be brought out from time to time.]

### BRIGGS' INDIAN FOOD.

(BRIGGS AND CO., 65 AND 66 CHANCERY LANE, LONDON, W.C.)

THIS prepared food is made from Indian cereals and contains all the nutritive properties of grain in an extremely assimilable form. It is finely divided, partially predigested, and highly suitable for invalids and growing children. It contains a high percentage of proteids, acids, salts, and, considering that it is a cereal preparation, a good percentage of fat. It is ready for use and does not require any additional cooking.

